# Comparison of Open Abdominal and Laparoscopic Bilateral Uterosacral Ligament Replacement: A One-Year Follow-Up Study

**DOI:** 10.3390/jcm14061880

**Published:** 2025-03-11

**Authors:** Sebastian Ludwig, Mathieu Pfleiderer, Jodok Püchel, Constanze Amir-Kabirian, Janice Jeschke, Dominik Ratiu, Christian Eichler, Bernd Morgenstern, Peter Mallmann, Julia Radosa, Fabinshy Thangarajah

**Affiliations:** 1 Department of Gynecology and Obstetrics, Faculty of Medicine, University Hospital Cologne, University of Cologne, Kerpener Straße 62, 50931 Cologne, Germany; mathiue.pfleiderer@uk-koeln.de (M.P.); jodko.puechel@uk-koeln.de (J.P.); constanze.amir-kabirian@uk-koeln.de (C.A.-K.); christian.eichler@uk-koeln.de (C.E.); bernd.morgenstern@uk-koeln.de (B.M.);; 2 Department of Gynecology and Obstetrics, Faculty of Medicine, University Hospital of Münster, University of Münster, 48149 Münster, Germany; janicekatharina.jeschke@ukmuenster.de; 3 Department of Obstetrics and Gynecology, University Hospital of Bern, University of Bern, 3010 Bern, Switzerland; nickblank@gmx.de; 4 Department for Gynecology, Obstetrics and Reproductive Medicine, Saarland University Hospital, 66421 Homburg, Germany; julia.radosa@uks.eu; 5 Department of Gynecology and Obstetrics, University Hospital of Essen, 45147 Essen, Germany; fabinshy.thangarajah@uk-essen.de

**Keywords:** pelvic organ prolapse, pelvic floor dysfunction, polyvinylidene fluoride, urinary incontinence, bilateral apical fixation, bilateral cervicosacropexy, bilateral sacropexy

## Abstract

**Background:** Pelvic organ prolapse significantly affects women’s health, often requiring surgery. Unilateral sacrocolpopexy (SCP) is the gold standard for apical prolapse repair. However, varied SCP techniques can lead to inconsistencies in clinical outcomes, with differences in synthetic materials, mesh dimensions, placement, and apical tensioning. This variability may impact the comparability of clinical outcomes. Bilateral apical fixation has gained attention for its potential to provide effective apical support and restore anatomical integrity. **Objective:** To date there are not many studies on bilateral apical cervicosacropexy between the vaginal apex and the sacrum at the level of S1/promontory with one-year follow-up. **Methods:** This study presents a one-year follow-up comparing the clinical outcomes of open abdominal (CESA) and laparoscopic cervicosacropexy (laCESA) for bilateral apical suspension in women with pelvic floor disorders. A total of 145 women underwent either CESA (*n* = 75) or laCESA (*n* = 70) using a surgical technique with a designed polyvinylidene-fluoride (PVDF) mesh of defined shape replacing both uterosacral ligaments. Outcomes were efficacy, safety, and success rates of both surgical approaches in restoring apical vaginal support and pelvic floor functioning. **Results:** Both techniques demonstrated high efficacy of apical prolapse repair and a high level of safety. While comparable rates of urinary continence restoration were achieved, laCESA showed significant advantages in terms of operative time, hospital stay, and recovery time. **Conclusions:** These findings demonstrate the reproducibility of a surgical technique including clinical outcomes in the treatment of pelvic floor dysfunction. The standardization of mesh design and surgical methodology enhances reproducibility and may mitigate some of the variability associated with clinical outcomes in apical mesh fixation techniques.

## 1. Introduction

Pelvic organ prolapse (POP) is a prevalent condition significantly impacting women’s health and quality of life [1]. Surgical intervention is frequently required, with unilateral sacrocolpopexy (SCP) considered the gold standard for apical POP repair due to its high success rates [2,3]. However, the heterogeneity of SCP techniques presents challenges for both clinical practice and research [4]. The absence of standardized protocols introduces significant variability in surgical approaches, encompassing the type of synthetic material employed, the mesh dimensions (size and shape), the precise placement of the mesh (unilateral), and the degree of apical tensioning applied during the surgical procedure [5,6]. This variability undermines the comparability and reproducibility of clinical outcomes across different studies [7,8].

Among the various surgical interventions, bilateral apical fixation has gained attention for its potential to provide effective support and restore anatomical integrity in women suffering from apical prolapse [8,9].

To address the limitations of heterogeneous apical fixation techniques, a novel surgical approach was developed [10,11,12,13]. This cervicosacropexy (CESA) employs a minimal amount of biocompatible synthetic material—specifically, polyvinylidene fluoride (PVDF)—to restore the physiological fixation of the vaginal apex through bilateral uterosacral ligament replacement [14]. This technique uses controlled bilateral apical traction, unlike many conventional techniques that rely on unilateral traction. Initially performed using an open abdominal surgical approach, CESA effectively addresses apical suspension, elevates the anterior vaginal wall, and through this, provides support for the bladder base and neck [10,12,15,16]. The use of a designed PVDF mesh of identical shape and dimensions further enhances the reproducibility of this technique, with the defined fixation points located between the cervical cut surface and the sacral vertebra at the level of S1/promontory [14].

The inherent advantages of minimally invasive surgery, particularly regarding reduced invasiveness and faster recovery, have prompted the adaptation of the CESA technique to a laparoscopic approach (laCESA) [12,13]. While preserving the core principles of CESA, the laCESA technique incorporates enhanced surgical access and instrumentation benefits.

Apart from the bilateral sacrospinous fixation, a lateral suspension and pectopexy, there are hardly any bilateral apical fixings of the vaginal apex at the sacrum at S1/promontory [3,17,18,19]. According to Delancey and colleagues, the effectiveness of an apical fixation on the promontory is highest [20,21,22]. So far there is only very limited literature that deals with bilateral apical cervicosacropexy between the vaginal apex and the sacrum at the level of S1/promontory [11,12,15]. This study aims to carry out a comprehensive one-year follow-up of a methodically well-defined bilateral apical suspension using various access routes. It tries to compare the effectiveness and safety of these approaches and at the same time ensure reproducibility and consistency in the clinical results and thus contributes significantly to the current data.

## 2. Methods

This retrospective observational study analyzed data from 145 women who underwent bilateral uterosacral ligament (USL) replacement using either open abdominal cervicosacropexy (CESA) or laparoscopic cervicosacropexy (laCESA) for apical prolapse repair with one-year follow-up. This study was conducted at the University Hospital of Cologne’s Department of Obstetrics and Gynecology, Division of Urogynecology, between June 2014 and May 2019. The established CESA technique was employed for all procedures [13].

### 2.1. Patient Inclusion and Exclusion Criteria

**Figure 1:**
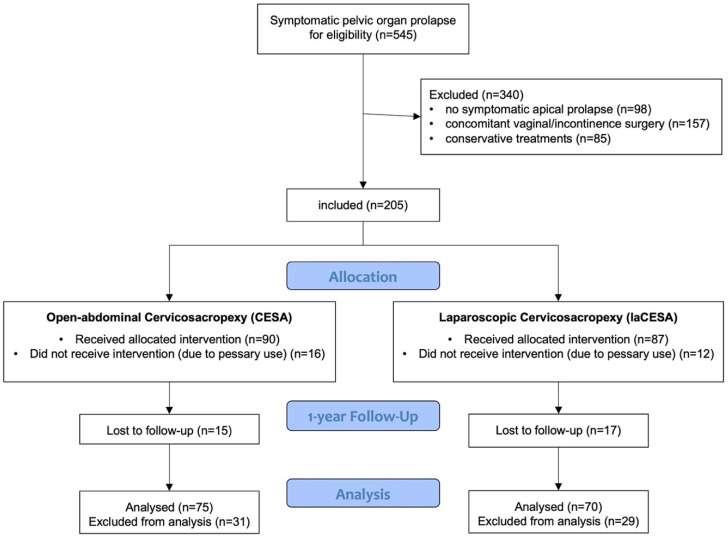
Flowchart of included patients and allocation.

During 2014 to 2019, a total of over 1600 patients presented to our tertiary university center with pelvic floor dysfunction. Of these, 517 patients had prolapse symptoms (with or without urinary incontinence). Of these 517 patients, 419 had symptomatic apical prolapse (POP-Q stage I–IV) of the uterus. In 205 patients, the sole indication was for surgical apical bilateral fixation (with or without supracervical hysterectomy) without concurrent vaginal or incontinence surgery. Thirty-two patients (16%) were lost to follow-up and a total of 28 patients (14%) refused surgery and preferred pessary therapy (Figure 1). This study included non-pregnant women with POP-Q stages I-IV of symptomatic apical prolapse, with or without urinary incontinence (UI), aged 18 years and older or previous surgery. Exclusion criteria were a body mass index (BMI) exceeding 55 kg/m^2^, the presence of pure stress urinary incontinence (SUI) without apical prolapse, and the absence of complete one-year postoperative follow-up data.

The retrospective design of this study was due to the fact that until April 2026 the cervicosacropexy was only performed via laparotomy. As of May 2016, the procedure was performed almost exclusively laparoscopically. Randomization was not planned here in order not to deprive the patients of the advantages of minimally invasive laparoscopy. All patients provided written informed consent prior to the study, and the study protocol received ethical approval from the Ethical Committee of the medical faculty of the University of Cologne on 13 February 2020 (Approval No. 20-1016). Consent was also obtained from participants for the publication of individual patient data. All methods were performed in accordance with the relevant national guidelines and regulations for the treatment of patients with pelvic floor dysfunctions. There was no funding for this study.

### 2.2. Preoperative and Postoperative Assessments

All patients underwent comprehensive preoperative and postoperative urogynecological examinations. These evaluations included a detailed history of prolapse and UI symptoms, a review of any previous gynecological or urogynecological surgeries and anti-incontinence procedures, and a baseline assessment of relevant clinical parameters (Table 1). Pelvic organ prolapse (POP) was objectively assessed using the Pelvic Organ Prolapse Quantification (POP-Q) system [23,24]. Complete prolapse resolution was defined as the absence of uterine prolapse at one year (apical POP-Q stage 0). UI symptoms were assessed based on patient-reported subjective experiences using validated questionnaires, specifically the International Consultation on Incontinence Questionnaire—Short Form (ICIQ-UI SF) [25]. Urodynamic studies were not routinely performed. UI was categorized as stress UI (SUI), urgency UI (UUI), or mixed UI (MUI) based on the patients’ responses in the ICIQ-UI SF. The majority of patients with UI had received prior medical or conservative treatment before surgical intervention. Patients were classified as continent if they remained free of UI symptoms at the one-year follow-up according to the above-mentioned questionnaires.

### 2.3. Outcome Measures

The primary outcome measures were the restoration of apical prolapse (defined as POP-Q stage 0) at the one-year follow-up. Secondary outcome measures were the complications of the procedure (related to mesh implantation) and included the following effects: pelvic floor functioning (urinary continence), operative time, postoperative hospital stay, and time to return to normal daily activities (documented at 2 and 16 weeks postoperatively).

### 2.4. Surgical Techniques

**Figure 2:**
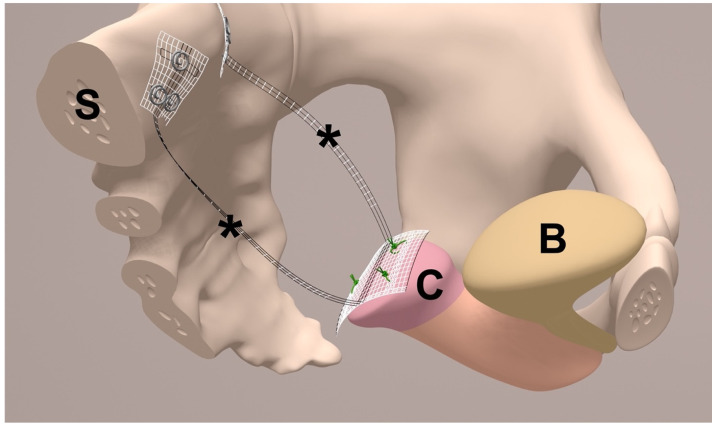
Half-sagittal view of bilateral uterosacral ligament (USL) replacement in the female small pelvis. The middle part of the polyvinylidene fluoride (PVDF) structure (*) is sutured to the cut surface of the cervix (*C*). The bladder (*B*) is not separated and remains on the cervix (*C*). The * indicate both arms of the PVDF structure (Dynamesh CESA, FEG Textiltechnik mbH, Aachen, Germany) for USL replacement. Each arm has a defined length of 8.8 cm and width of 0.4 cm and is placed below the peritoneal fold of the left and right USL. Both ends of the PVDF structure are fixed in front of the sacral vertebra (*S*) to the prevertebral fascial layer at the level of S1/promontory (made with Adobe Illustrator 2021, Version 25.2.3, Dublin, Republic of Ireland).

**Figure 3:**
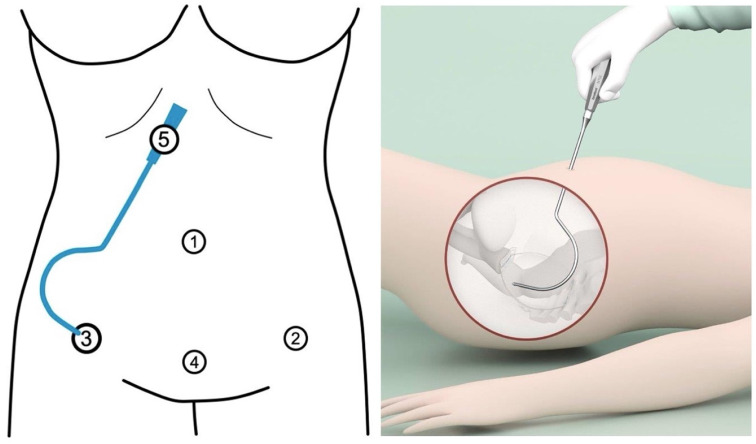
Trocar placement and insertion of uterosacral ligament (USL) tunneling device. Port locations with 4 trocar entries in laparoscopic cervicosacropexy: Opitcal, umbilicus (10 mm) (*1*). Left lower abdomen, in the anterior axillary line at the level of superior spina ischiadica, laterally to the epigastric vessels (5 mm) (*2*). Right lower abdomen, in the anterior axillary line at the level of superior spina ischiadica, laterally to the epigastric vessels (5 mm) (*3*). Centrally placed trocar in the lower abdomen, within the middle line 2 cm above the symphysis (*4*). As a tunneling device (*5*), a semi-circular curved hook (IVT02, DynaMesh, FEG) with a handle is used. The right lateral trocar incision (*3*) is used for USL tunneling: after the right lateral trocar has been removed, the tunneling device is inserted through its incision (personal collection) (made with Adobe Illustrator 2021, Version 25.2.3, Dublin, Ireland).

**Figure 4:**
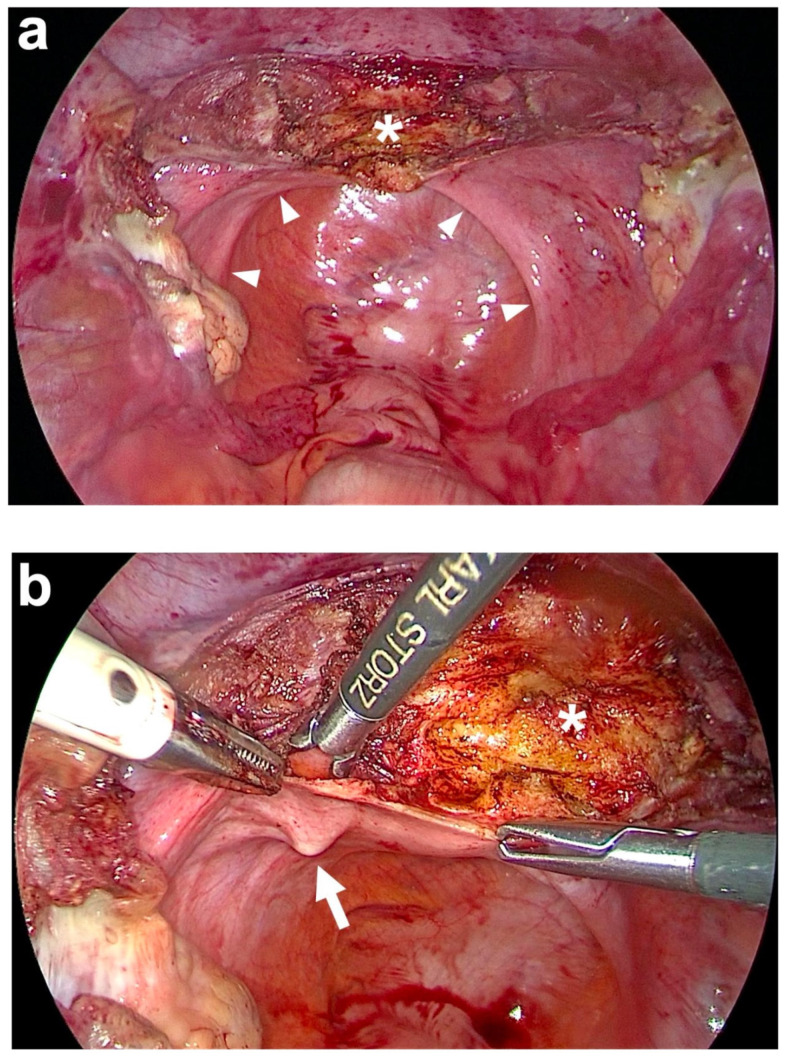
Subtotal hysterectomy and preparation of the anterior fixation side. (**a**) After subtotal hysterectomy, the cut surface of the cervix (white asterisk) is seen. The uterus is cut above the attachments of both uterosacral ligaments (USL) and at the beginning of the bladder peritoneum on the anterior cervix using a monopolar electric needle. The remnants of both USL are marked on the left and right in the small pelvis by white arrowheads. (**b**) Preparation of the left USL origin (white arrow) at the paracervical tissue left to the cervix (white asterisk). The same is performed on the right side (personal collection).

**Figure 5:**
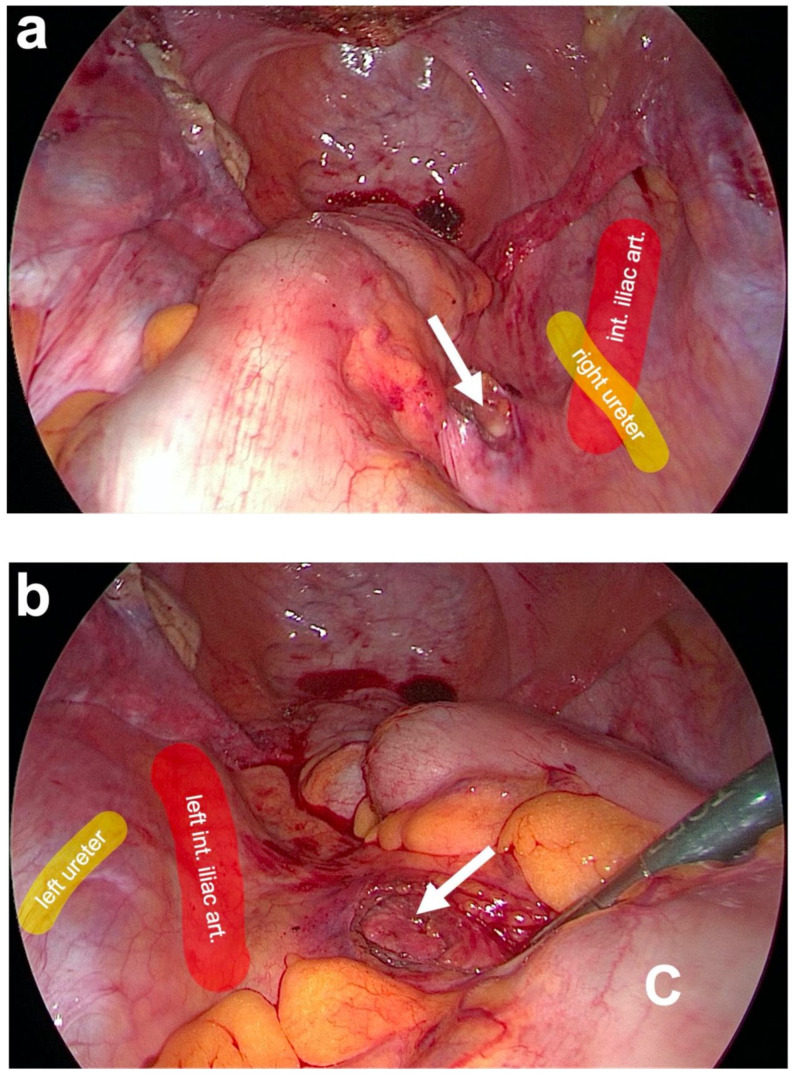
Preparation of posterior fixation sides at the promontory. (**a**) Blunt dissection of the prevertebral fascial layer on the right side of the promontory (white arrow). Here, the right ureter and the right iliac vessels are lateral to the dissection site (as partially marked in color). (**b**) After pulling aside the descending colon (C) with a clamp, the blunt dissection of the prevertebral fascial layer on the left side of the promontory (white arrow) is carried out. The left ureter and the left iliac vessels are lateral to the dissection site (as partially marked in color) (personal collection).

**Figure 6:**
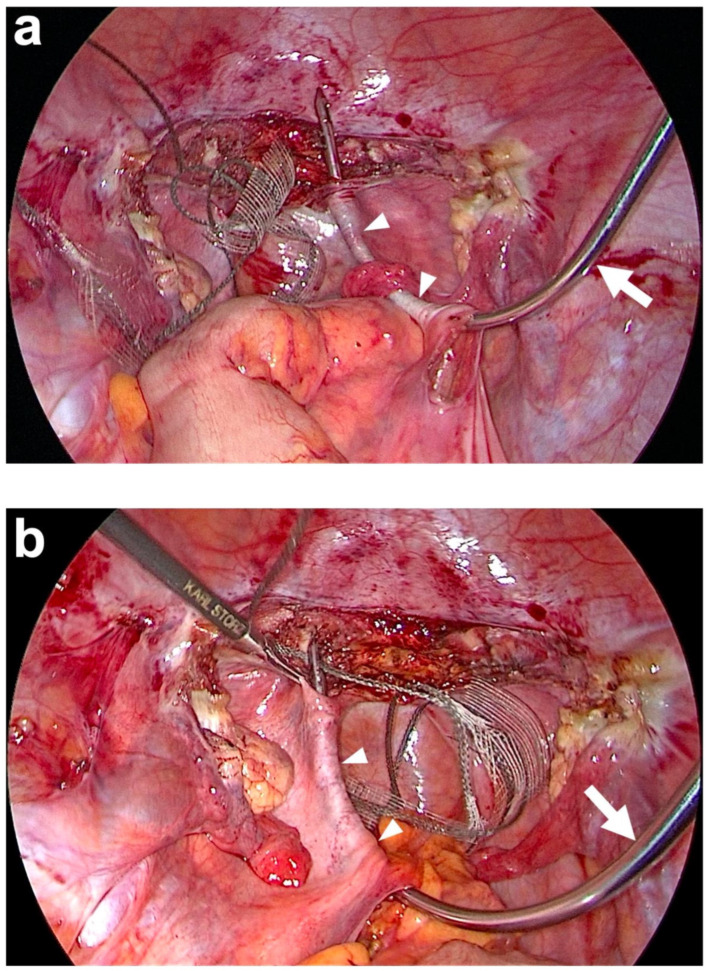
Tunneling of both uterosacral ligaments (USL) (white arrowheads) with the tunneling device (semi-curved hook). (**a**) The tunneling device (white arrow) is brought into the abdominal cavity via the right lateral trocar incision and then inserted through the peritoneal window previously prepared on the promontory on the right. The blunt tip of the tunneling device is then carefully advanced below the peritoneum paracervically to the right until it is exposed. The end of the right PVDF structure is now attached to the tip of the tunneling device and inserted into the right course of the original USL as it is retracted. (**b**) The same procedure on the left side after the colon has been pulled to the side. The tunneling device is inserted through the peritoneal window previously prepared on the promontory on the left. The blunt tip of the tunneling device is then carefully advanced below the peritoneum paracervically on the left until it is exposed. The end of the left PVDF structure is attached to the tip of the tunneling device and inserted into the left course of the original USL as it is retracted (personal collection).

**Figure 7:**
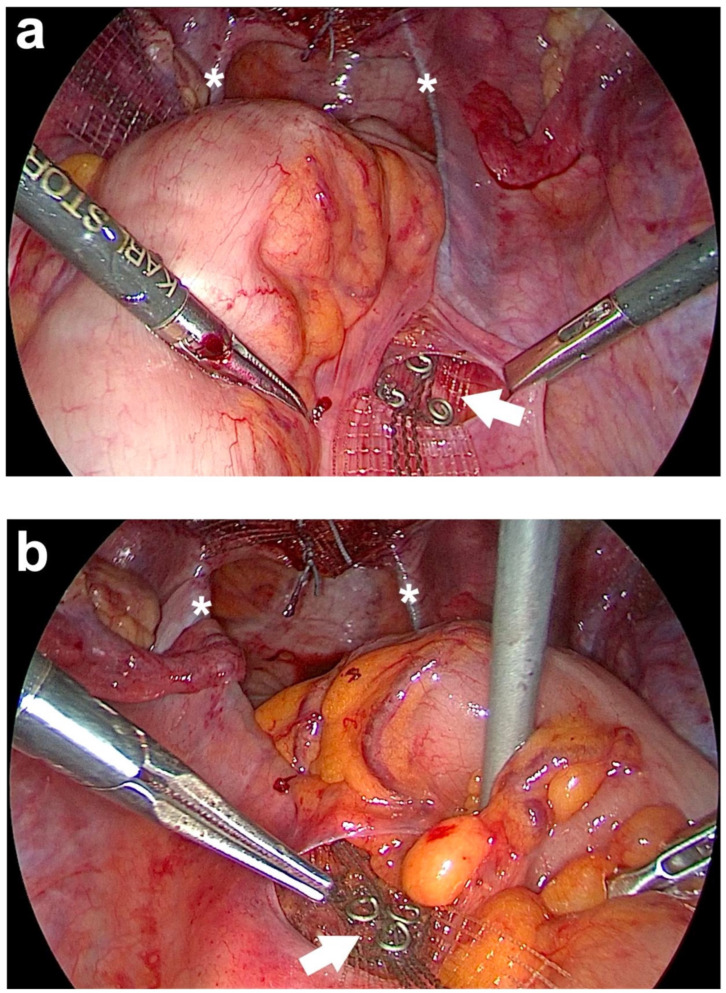
Posterior fixation of both arms of the PVDF (polyvinylidene fluoride) structure for uterosacral ligament (USL) replacement left and right at the sacrum. (**a**) Fixation of the right arm of the PVDF structure in the designated markings using three titanium helices (white arrow). (**b**) Fixation of the left arm of the PVDF structure in the designated markings (white arrow). The white asterisks show both PVDF structures, which lie in the original course of both USLs. After fixing the band structures, the ends of these two are cut off cranially of the helices (personal collection).

**Figure 8:**
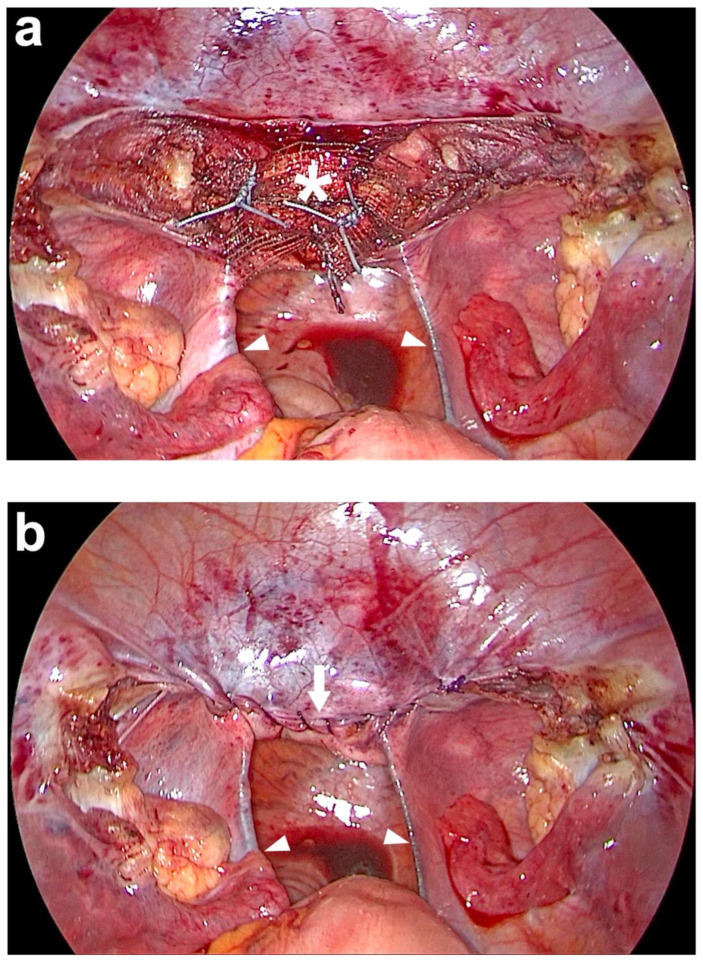
Peritoneal closure above the middle part of the PVDF (polyvinylidene fluoride) structure on the cut surface of the cervix. (**a**) The PVDF structure (white asterisk) is sutured to the cut surface of the cervix with 3 interrupted, non-resorbable sutures. (**b**) At the end of the laparoscopy, the peritoneum above the middle part of the PVDF (polyvinylidene fluoride) structure on the cut surface of the cervix is closed with a running, absorbable suture (white arrow). The entire PVDF structure is covered by peritoneum, including both arms which lie in the original course of both uterosacral ligaments (USL) (white arrowheads). Due to the bilateral course in the peritoneal fold of the USL and the posterior attachment left and right laterally to the sacrum, the intestine is not constricted (personal collection).

To achieve apical suspension, a bilateral cervicosacropexy was used [13]. This cervicosacropexy was performed either open abdominally (CESA) or laparoscopically (laCESA), utilizing a commercially available polyvinylidene fluoride (PVDF) mesh (Dynamesh CESA, FEG Textiltechnik mbH Company, Aachen, Germany) of identical dimensions (8.8 cm length and 0.4 cm width) (Figure 2) [14]. The mesh was strategically placed to replace both USL below the peritoneal fold between the cervix and the sacrum at the level of S1/promontory. The central portion (2 × 4 cm) of the PVDF structure was sutured to the cut surface of the cervix, while the distal ends (1 × 2 cm) served as fixation points at the sacral vertebra (Figure 2). A semi-circular curved tunneling device (IVT02, DynaMesh, FEG Textiltechnik mbH, Aachen, Germany) facilitated the placement of the PVDF mesh within the peritoneal folds of both USL (0.4 cm width), maintaining peritoneal integrity (Figure 3, Figure 4, Figure 5, Figure 6, Figure 7 and Figure 8 illustrate the steps of the laCESA technique). In CESA (open procedure), the PVDF structure was fixed at the S1 level using two non-absorbable sutures, in laCESA (laparoscopic), fixation occurred at the S1/promontory using three titanium helices for each side (ProTack, Covidien, Mansfield, MA, USA) (Table 2 provides a detailed summary of the surgical steps). The animated figures were made with Adobe Illustrator 2021, Version 25.2.3, Dublin, Republic of Ireland. The intraoperative images/figures belong to the corresponding author’s personal collection.

### 2.5. Statistical Analysis

Patient data were managed using MS Excel 2011 (Microsoft Corporation, Redmond, WA, USA). Frequencies were presented as percentages, while continuous data were expressed as means ± standard deviations and medians. Statistical comparisons between CESA and laCESA groups were performed using appropriate parametric and non-parametric methods (Mann–Whitney U test, Pearson’s chi-squared test, *t*-tests, and Fisher’s exact test). All *p*-values were two-sided, and statistical significance was set at *p* < 0.05. Statistical analyses were conducted using SPSS version 22 (IBM, Armonk, NY, USA).

## 3. Results

This retrospective study included 145 women with varying degrees of symptomatic apical prolapse and urinary incontinence (UI). Baseline demographic and clinical characteristics of the study population, including age, BMI, parity, details of prolapse staging according to the Pelvic Organ Prolapse Quantification (POP-Q) system, and preoperative UI status, are summarized in Table 1. Of the 145 participants, 75 (52%) underwent open abdominal cervicosacropexy (CESA), while 70 (48%) underwent laparoscopic cervicosacropexy (laCESA).

### 3.1. Preoperative Characteristics

Analysis of the baseline clinical parameters revealed that, within the analyzed study cohort, 56 patients (39%) presented with symptomatic POP-Q stage 1 apical prolapse, 73 patients (50%) presented with POP-Q stage 2 apical prolapse, and 16 patients (11%) presented with POP-Q stages 3–4 apical prolapse. All 205 patients included in this study were offered pessary therapy before surgery. Of these 205 patients, 29 used a pessary for a short period of time but still wanted to undergo surgery. A total of 28 patients (14%) refused surgery and preferred pessary therapy. A total of 32 patients did not return for follow-up and were excluded from the analysis, as were the 28 patients with the pessary, so that 145 patients with complete follow-up 1 year after surgery could be evaluated.

A substantial majority of patients (106 out of 145; 73%) experienced UI preoperatively. Patients with apical POP-Q stage 1 and UI received conservative management, including anticholinergic medication (28 out of 106; 26%) or anti-incontinence surgical procedures (6 out of 106; 6%) as indicated. No statistically significant differences in these baseline clinical parameters were observed between the CESA and laCESA groups (Table 1). A total of 24 patients (17%) reported a history of prior urogynecological surgeries.

### 3.2. Operative and Postoperative Findings

The mean operative time for abdominal CESA was 120 min (range: 89–168 min); whereas, for laCESA, it was 93 min (range: 58–137 min). However, it is important to note that, when considering the learning curve effect associated with the introduction of the laparoscopic technique, the average operative time for the last 10 laCESA procedures was significantly reduced to 89 min. None of the laparoscopic procedures necessitated conversion to an open abdominal approach (Table 3).

Postoperative hospital stays were also shorter for the laCESA group (mean 3 days; range: 1–5 days) compared to the CESA group (mean 5 days; range: 3–8 days). Furthermore, patients reported a significantly faster return to their usual daily activities following laparoscopy (1 week) compared to the open-abdominal procedure (3 weeks) (Table 3).

### 3.3. Intraoperative and Postoperative Complications

No major intraoperative complications, such as ureteral or bowel injury, or vascular injury requiring a blood transfusion, were observed in either surgical group (Table 3). Minor complications were observed more frequently in the CESA group. These minor complications included one case of bowel serosal lesion due to adhesion formation, one case of bladder lesion related to adhesions following a prior cesarean section, and two instances of postoperative bowel obstruction requiring prolongation of hospitalization. In the laCESA group, one patient experienced injury to the epigastric vessels during trocar placement, and another sustained a bladder lesion during trocar insertion. Importantly, no cases of mesh erosion were detected in either surgical group during the follow-up period. At one year postoperatively, no cases of apical prolapse recurrence were observed. One patient in the CESA group required revision surgery (re-laparoscopy and cervical amputation) due to inadequate cervical fixation resulting from an incomplete supracervical hysterectomy and an elongated anterior uterine orifice. Additional procedures were required in some patients postoperatively: 20 out of 87 (23%) in the CESA group required an anterior colporrhaphy for symptomatic cystocele, while 2 out of 15 (13%) in the laCESA group required a posterior colporrhaphy for symptomatic rectocele. These data are detailed in Table 3.

### 3.4. Urinary Continence Outcomes

Preoperative UI was present in 106 patients (73%), with 58 in the CESA group and 48 in the laCESA group. A higher proportion of patients in the CESA group presented with mixed urinary incontinence (MUI) preoperatively (Table 1). At four months postoperatively, 24 out of 58 (41%) patients in the CESA group and 21 out of 48 (43%) patients in the laCESA group reported urinary continence. No instances of *de novo* urgency were observed.

Higher urinary continence rates were observed in patients younger than 60 years of age compared to older cohorts. Specifically, among the younger demographic, the continence rate following cervicosacropexy (CESA) was 64%, whereas for laparoscopic cervicosacropexy (laCESA) it was 68%. There were no significant differences between the two surgical methods. Conversely, in patients aged 60 years and older, the urinary continence rates were notably lower, with 34% for CESA and 37% for laCESA. Furthermore, no further significant difference in the variables was found with regard to predictive factors such as patient characteristics, surgical procedure/techniques, or complications.

At the one-year follow-up, an additional 21 transobturator tapes (TOTs) were placed in the CESA group, resulting in a 59% continence rate (34 out of 58 patients) [26]. In the laCESA group, 18 TOTs were placed, leading to a 60% continence rate (29 out of 48 patients). It should be noted that there were no de novo SUI and the TOTs were placed in patients who initially presented with MUI (Table 4).

In addition, 22 patients (15%) required additional vaginal repairs (colporrhaphy for cystocele or rectocele), and two patients (1%) had an additional colposuspension (Table 4).

## 4. Discussion

A variety of surgical techniques exist for apical suspension, aiming to restore anatomical support and, in many cases, improve pelvic floor functioning [4,8,9]. Sacrocolpopexy (SCP) is widely regarded as the gold standard for surgical apical repair [3,27]; however, its clinical application is often hampered by considerable variability in surgical approaches. This variability arises from surgeon-dependent decisions concerning mesh type, size, shape, placement, and the degree of apical tensioning [5,6,7]. The inconsistent application of these parameters may lead to considerable methodological heterogeneity across clinical studies [9,25,26]. This heterogeneity makes the direct comparison of clinical outcomes challenging and limits the reproducibility of results [6,9]. Apart from the bilateral sacrospinous fixation, a lateral suspension or pectopexy, there are hardly any bilateral apical fixations of the vaginal apex at the sacrum at the level of S1/promontory [3,17,18,19]. According to Delancey and colleagues, the effectiveness of an apical fixation on the promontory is highest [20,21,22]. So far, there is only very limited literature that deals with bilateral apical fixation between the vaginal apex and the sacrum at the level of S1/promontory [11,12,13].

To overcome these limitations inherent in existing unilateral SCP techniques, the bilateral cervicosacropexy (CESA) technique was developed as described [12,13,28]. This bilateral apical suspension prioritizes the re-establishment of the physiological fixation of the cervix, thereby supporting the vagina and anterior vaginal wall [20,21,22,29,30]. This is accomplished by replacing both uterosacral ligaments (USLs) with a minimal amount of biocompatible synthetic material: specifically, polyvinylidene fluoride (PVDF) mesh of identical length (8.8 cm) and width (0.4 cm) [13]. Initially conceived as an open abdominal procedure, the CESA technique was subsequently adapted for laparoscopic surgery (laCESA) due to the recognized advantages of minimally invasive surgery, such as reduced invasiveness, smaller incisions, decreased postoperative pain, and faster recovery [10,12,13,15,28].

The adaptation of CESA to the laparoscopic approach necessitated minor modifications and adjustments in surgical access and instrumentation. To facilitate retroperitoneal tunneling of both USLs during laCESA, a specialized curved tunneling device was employed [13]. In traditional laparoscopic procedures, instruments are typically straight, as they are inserted through rigid trocars. However, these straight instruments can be cumbersome when placing the USL structure into the semi-circular peritoneal fold of the USLs. To overcome this challenge, we chose to use a semi-circular tunneling device that can be conveniently inserted through a lateral trocar incision (Figure 3).

Once the tip of the tunneling device is positioned within the peritoneal folds of the USLs, it glides beneath the peritoneum towards the lateral cervix on both sides of the small pelvis. This technique significantly reduces the risk of major injuries to surrounding vessels, ureters, and nerves and thus the peritoneum is left intact [12,13]. While minor adjustments were necessary to optimize the laCESA technique, these modifications did not introduce significant complications such as bowel perforation, vascular injury, or ureteral lesions.

Despite these technical progress and standardized surgical procedures, the intraoperative assessment of the anatomical structures and the implanted meshes in the context of prolapse surgeries remains a challenge [31]. In this context, intraoperative ultrasound diagnostics are becoming increasingly important since they enable direct and dynamic visualization of the operated structures [32]. For example, after mesh implantation and fixation, a Doppler-ultrasound or organ ultrasound of the corresponding anatomical structures can be carried out intraoperatively in order to prevent ureteral kinking or vascular injury and possible later consequences. In the future of prospective studies, this aspect should find significantly more attention in the future.

Moreover, the use of PVDF meshes, particularly when compared to polypropylene meshes, offers superior biocompatibility and reduced adhesion formation [33,34,35,36]. For example, the smallest mesh area described in the literature for a unilateral sacrocolpopexy is 45 cm^2^ [5]. In comparison to the PVDF tapes used here, only an area of about 16 cm^2^ is implanted, and that is bilateral. This, combined with the minimal amount of synthetic material and the frequent use of supracervical hysterectomies, significantly minimizes the risk of mesh erosion [36]. The surgical technique, incorporating the use of PVDF tapes and meticulously defined fixation points, significantly enhanced the reproducibility of results.

The retrospective design of this study has inherent limitations, primarily regarding the potential for selection bias and the inability to establish definitive cause-and-effect relationships. Initially, of the potential 545 patients who presented to our tertiary center during this period, we had to exclude 340 from the current analysis. To isolate the effect of bilateral apical suspension and compare the two surgical methods, we included only patients with symptomatic apical prolapse who did not undergo concomitant vaginal or anti-incontinence surgeries. Additionally, patients who were treated solely with conservative measures were also excluded (Figure 1). The decision to perform most of the apical fixations using the laCESA technique from 2016 onwards reflects our commitment to providing patients with the benefits of minimally invasive surgery. The retrospective study design, incorporating patients undergoing CESA prior to 2016, allowed us to collect sufficient data to compare the one-year outcomes of both surgical techniques. A larger-scale, prospective, randomized controlled trial (RCT) would provide more robust evidence to further support these findings. Furthermore, incorporating validated quality-of-life questionnaires would offer a more comprehensive assessment of patient outcomes, including the impact of each surgical approach on patient experience and functional well-being.

Despite these limitations, the current study’s strengths lie in its detailed data collection, the exact specified surgical technique with a methodological homogeneity, and utilization of validated assessment tools such as the POP-Q system and validated UI questionnaires, thus enhancing the reliability and interpretability of the results.

These findings show the reproducibility of a surgical technique between laparotomy and laparoscopy. In a comprehensive review by de Boer et al., various prolapse surgeries were examined, highlighting not only the correction of prolapse but also the restoration of urinary continence [8]. Specifically, the healing rates for the restoration of urinary continence following prolapse surgeries in the cited publication ranged from 4% to 80%. This review encompasses all types of prolapse surgeries, including vaginal procedures such as anterior and posterior repairs with or without mesh, vaginal hysterectomy, Kelly stitches, and Manchester repair, as well as sacrocolpopexy and apical suspension. When focusing solely on sacrocolpopexies and apical suspensions, the healing rates vary between 11.9% and 30.9% [8].

These differences in continence rates further underscore the heterogeneity of apical suspension techniques. In the present study, despite the identical correction of prolapse (100% after CESA and laCESA), there is no significant difference observed in continence rates when comparing the open abdominal and laparoscopic approaches (59% vs. 60%).

## 5. Conclusions

In conclusion, the introduction of cervicosacropexy, employing controlled bilateral traction and the use of biocompatible PVDF material, presents a significant advancement in prolapse surgery and pelvic floor functioning. It appears to be one safe and effective short-term (one-year follow-up) treatment option. These results demonstrate the reproducibility of a surgical technique between two different abdominal access routes (CESA and laCESA). Additionally, this procedure is associated with significant improvements in pelvic floor functioning, especially in UI. While the specific factors that influence the pathogenesis of the UI remain unclear, it turned out that age at surgery played a critical role (especially over 60 years). In the under 60 years old, there is a higher continence rate after CESA and laCESA.

The standardization of mesh design and surgical methodology enhances reproducibility and may mitigate some of the variability associated with surgical outcomes in traditional unilateral apical mesh fixation techniques. Future prospective, randomized controlled trials should be undertaken to further investigate the long-term efficacy, safety, and patient outcome of bilateral apical mesh fixation.

## Figures and Tables

**Table 1:** Baseline characteristics of the 145 patients before cervicosacropexy, divided into 2 abdominal (CESA) and laparoscopic (laCESA) surgical techniques.

Characteristic	CESA ^a^ (*n* = 75)	laCESA ^a^ (*n* = 70)	*p*-Value
Age, mean years (range)	59 (32–82)	58 (28–81)	0.714 ^b^
Body mass index (BMI) ^c^	27 (18–42)	26 (18–37)	0.215 ^b^
Parity, mean (range)	2 (0–7)	2 (0–5)	0.467 ^b^
Pelvic organ prolapse ^d^			
apical POP-Q stage 0	0 (0%)	0 (0%)	1.000 ^e^
apical POP-Q stage 1	33 (44%)	23 (33%)	0.177 ^e^
apical POP-Q stage 2	37 (49%)	36 (51%)	0.869 ^e^
apical POP-Q stage 3–4	5 (7%)	11 (16%)	0.112 ^e^
Type of urinary incontinence ^f^			
Urinary incontinent	58 (77%)	48 (69%)	0.264 ^e^
Stress urinary incontinence (SUI)	7 (12%)	8 (17%)	0.788 ^e^
Urgency urinary incontinence (UUI)	11 (19%)	12 (25%)	1.000 ^e^
Mixed urinary incontinence (MUI)	40 (69%)	28 (28%)	0.021 ^e^
History of previous surgery ^g^			
Sacrocolpopexy	2 (3%) ^h^	0 (0%)	0.170 ^e^
Laparoscopic subtotal hysterectomy	6 (8%) ^i^	5 (7%)	0.846 ^e^
Anterior colporrhaphy	3 (4%)	1 (1%)	0.602 ^e^
Anterior colporrhaphy with mesh	0 (0%)	1 (1%)	0.301 ^e^
Colposuspension (Burch)	1 (1%)	0 (0%)	0.334 ^e^
Tension-free vaginal tape (TVT)	4 (5%)	0 (0%)	0.092 ^e^
Transobturator tape insertion (TOT)	1 (1%)	0 (0%)	0.334 ^e^

^a^ Values are given as number of all patients (percentage) unless indicated otherwise; ^b^ *t*-test; ^c^ values calculated as weight in kilograms divided by the square of height in meters, values are given as mean (range); ^d^ apical prolapse according to the Pelvic Organ Quantification (POP-Q) system; ^e^ Pearson’s chi-squared test; ^f^ according to validated urinary incontinence questionnaires; ^g^ prior urogynecological surgeries; ^h^ insufficient sacrocolpopexy; ^i^ in one patient an abdominal subtotal hysterectomy was performed instead of laparoscopic.

**Table 2:** Differences in the surgical steps and surgical approaches between the open abdominal and laparoscopic cervicosacropexy (CESA) for bilateral uterosacral ligament (USL) replacement.

Surgical Steps	Abdominal CESA	Laparoscopic CESA
Surgical access path	Pfannenstiel incision	Establishment of CO_2_ peritoneum ^a^ Four trocars (Figure 3): − umbilical (10 mm) − left lower abdomen (5 mm) ^b^ − right lower abdomen (5 mm) ^b,c^ − at symphysis (10 mm) ^d^
Preparation of anterior fixation sides	Subtotal hysterectomy (if necessary) with monopolar electric knife above the origin of both USL and at peritoneal fold of bladder’s peritoneum at anterior cervix.	Subtotal hysterectomy (if necessary) with monopolar electric needle above the origin of both USL and at peritoneal fold of bladder’s peritoneum at anterior cervix (Figure 4a).
Anterior fixation of middle part of PVDF structure ^e^ (Figure 1)	Sutured to the cut surface of the cervix with 4 interrupted, non-absorbable sutures ^f^.	Sutured to the cut surface of the cervix with 3 interrupted, non-absorbable sutures ^f^ (Figure 8a).
Instrument for USL replacement (tunneling)	Semi-circular curved hook with blunt tip (tunneling device).	Semi-circular curved hook with blunt tip (tunneling device), inserted via the right lateral trocar incision (after removing the right trocar) (Figure 3).
Tunneling of both USL	The blunt tip of tunneling device is inserted into the left sacral peritoneal window and advanced under the peritoneum towards left paracervical tissue. Threading one lateral end of the PVDF structure through the hole of the tunneling device’s tip and pulling it back. Same procedure on the right side.	The blunt tip of tunneling device is inserted into the left sacral peritoneal window and advanced under the peritoneum towards the left paracervical tissue. Threading one lateral end of the PVDF structure through the hole of the tunneling device’s tip and pulling it back. Same procedure on the right side (Figure 6).
Preparation of posterior fixation sides	Incision of lateral peritoneum above S1/promontory for 2 cm on either side of rectosigmoid colon.	Incision of lateral peritoneum above S1/promontory for 2 cm on either side of rectosigmoid colon (Figure 5).
Posterior fixation of left and right arm of PVDF structure (Figure 1)	At left and right prevertebral fascial layer at S1 with 2 interrupted, non-absorbable sutures each ^e^ within the defined locations at the PVDF structure.	At left and right prevertebral fascial layer on S1/promontory with 3 titanium helices each ^g^ within the defined locations at the PVDF structure (Figure 2 and Figure 7).
Peritoneal closure	Closure of peritoneum above cut surface of cervix with running, absorbable suture ^h^.	Closure of peritoneum above cut surface of cervix with running, absorbable suture ^i^ (Figure 8b).

Cervicosacropexy, CESA; uterosacral ligaments, USL; polyvinylidene fluoride, PVDF; ^a^ according to institutional standards; ^b^ in the anterior axillary line at the level of superior spina ischiadica, laterally to the epigastric vessels; ^c^ used for “tunneling” of the USL’s peritoneal folds on both sides of the small pelvis (insertion of the peritoneal tunneling device after removal of the trocar); ^d^ within the middle line and 2 cm above symphysis; ^e^ DynaMesh CESA, FEG Textiltechnik mbH, Aachen, Germany; ^f^ non-absorbable sutures, Ethibond; Ethicon, Someville, NJ, USA; ^g^ fixation device, ProTack, Covidien, Mansfield, MA, USA; ^h^ absorbable suture, Vicryl 2-0, Ethicon, Johnson & Johnson Medical N.V., Machelen, Belgium; ^i^ running, absorbable suture (Quill, absorbable PDO, Surgical Specialities Corp., Westwood, MA, USA.

**Table 3:** Operative details and complications of the 145 patients operated on either by abdominal cervicosacropexy (CESA) or laparoscopic cervicosacropexy (laCESA).

Variable	CESA ^a^ (*n* = 75)	laCESA ^a^ (*n* = 70)	*p*-Value
Concomitant vaginal surgery, *n* (%)			
Anterior colporrhaphy	10 (13%)	18 (26%)	0.091 ^b^
Posterior colporrhaphy	1 (1%)	7 (10%)	0.029 ^b^
Transobturator tape insertion	0 (0%)	2 (3%)	0.231 ^b^
Colposuspension	2 (3%)	0 (0%)	0.497 ^b^
Vaginal surgery within follow-up, *n* (%)			
Transobturator tape insertion	21 (28%)	18 (26%)	0.852 ^b^
Anterior colporrhaphy	11 15%)	9 (13%)	0.813 ^b^
Posterior colporrhaphy	1 (1%)	1 (1%)	n.s. ^b^
Colposuspension	2 (3%)	0 (0%)	0.497 ^b^
Operating time (min), mean (range)	120 (89–168)	93 (58–137) ^c^	0.001 ^d^
Hospitalization (days), mean (range)	5 (3–8)	3 (1–5)	0.001 ^d^
Intraoperative complications, *n* (%)			
Bladder injuries ^e^	1 (1%)	1 (1%)	n.s. ^b^
Bowel injury	1 (1%) ^f^	0 (0%)	n.s. ^b^
Significant bleeding/Vessel injury	0 (0%)	0 (0%)	-
Ureter lesion	0 (0%)	0 (0%)	-
Complications at 1-year postoperative, *n* (%)			
Obstructed defecation	0 (0%)	0 (0%)	-
Reoperation for apical prolapse	0 (0%)	1 (1%) ^g^	0.483 ^b^
Mesh erosion	0 (0%)	0 (0%)	-
Urinary retention	0 (0%)	0 (0%)	-

^a^ Values are given as number of all patients (percentage) unless indicated otherwise; ^b^ Fischer’s exact test; ^c^ mean operation time excluding the first 10 patients (learning curve); mean operation time of the first 10 patients 117 min (102–137); ^d^ Mann-Whitney U test; ^e^ in one case bladder injuries during trocar placement and in the other case due to adhesions after caesarean section. Appropriate suturing in two layers and leaving an indwelling catheter for 10 days; ^f^ patient with severe adhesion formation of the bowl after prior laparotomy and serosa lesion of the sigma rectum without opening of the intestinal lumen; ^g^ first patient operated on via laparoscopic CESA had a cervical elongation, and cervical amputation was performed and re-laparoscopy was performed within follow-up; n.s., not significant.

**Table 4:** Clinical outcome one year after abdominal cervicosacropexy (CESA) and laparoscopic cervicosacropexy (laCESA).

Clinical Outcome	CESA (*n* = 75)	laCESA (*n* = 70)	*p*-Value
Pelvic organ prolapse ^a^, *n* (%)			
apical POP-Q stage 0	75 (100%)	70 (100%) ^b^	0.483 ^c^
apical POP-Q stage 1	0 (0%)	0 (0%)	-
Urinary continence status			
continent	34 (59%) ^d^	29 (60%) ^e^	0.726 ^c^
stress urinary incontinence	6 (10%)	8 (17%)	0.084 ^c^
urgency urinary incontinence	6 (10%)	3 (6%)	0.496 ^c^
mixed urinary incontinence	12 (21%)	8 (17%)	0.643 ^c^
ICIQ-UI score, mean (range) ^f^	3 (0–21)	4 (0–16)	0.704 ^g^

^a^ Apical prolapse according to the Pelvic Organ Quantification (POP-Q) system; ^b^ One patient had an insufficient cervical fixation (elongated anterior uterine orifice due to inadequate supracervical hysterectomy) and received re-laparoscopy and cervical amputation within 5 months after surgery; ^c^ Fischer’s exact test; ^d^ within one year after CESA, 21 transobturator tapes were additionally placed in incontinent patients; ^e^ within one year after laCESA, 18 transobturator tapes were additionally placed in incontinent patients; ^f^ ICIQ-UI SF, International Consultation on Incontinence Questionnaire—Short Form; ^g^ *t*-test.

## Data Availability

The datasets used and analyzed during the current study are not publicly available because they contain sensitive patient data, but they are available from the corresponding author in an anonymized and de-identified form upon reasonable request.

## Abbreviations

CESA	Cervicosacropexy
ICIQ-UI SF	International Consultation on Incontinence Questionnaire—Urinary Incontinence
laCESA	Laparoscopic cervicosacropexy
MUI	Mixed urinary incontinence
POP	Pelvic organ prolapse
POP-Q	Pelvic organ prolapse quantification system
PVDF	Polyvinylidene-fluoride
UI	Urinary incontinence
UUI	Urgency urinary incontinence
USL	Uterosacral ligament
SCP	Sacrocolpopexy
SUI	Stress urinary incontinence
TOT	Transobturator tape
